# Resolving Morphological Ambiguity in Molar Pregnancy: The Diagnostic Role of p57 Immunohistochemistry

**DOI:** 10.7759/cureus.110606

**Published:** 2026-06-10

**Authors:** Ananya Nath, Savitri M Nerune, Sayandeep K Das

**Affiliations:** 1 Pathology, Shri B. M. Patil Medical College, Vijayapura, IND

**Keywords:** complete mole, hydatidiform mole, non-molar pregnancy, p57 immunohistochemistry, partial mole

## Abstract

Objective: To evaluate the diagnostic utility of p57 immunohistochemistry (IHC) in differentiating complete and partial hydatidiform mole (PHM) from non-molar pregnancies, with emphasis on resolving morphologically ambiguous cases.

Materials and methods: This hospital-based cross-sectional study was conducted in a tertiary referral centre from March 2024 to October 2025. A total of 60 histopathologically diagnosed cases of complete hydatidiform mole (CHM), PHM, and non-molar pregnancies were included. Tissue sections were examined on H&E staining for morphological features, followed by p57 IHC on paraffin-embedded sections. Nuclear staining in villous cytotrophoblasts and stromal cells was evaluated. Statistical analysis was performed using appropriate tests, with p < 0.05 considered significant.

Results: Of 60 cases, 30 were non-molar and 30 molar pregnancies (12 complete, 14 partial, four unclassified on H&E). All non-molar cases showed positive p57 expression. Among CHMs, 91.7% were p57 negative, while 78.6% of PHMs were positive. Notably, 21.4% of cases initially diagnosed as PHMs lacked p57 expression and were subsequently categorised as CHMs based on combined histomorphological evaluation and immunohistochemical findings. Overall, p57 IHC led to reclassification in 13.3% of cases, mainly involving unclassified and PHMs. A significant association was observed between diagnosis and p57 expression (p < 0.001), driven by non-molar positivity and CHM negativity.

Conclusion: p57 IHC is a valuable adjunct to routine histopathology for differentiating CHM from PHM. Its use significantly improves diagnostic accuracy, particularly in early gestation and morphologically ambiguous cases, thereby facilitating appropriate clinical management, follow-up, and patient counselling.

## Introduction

Hydatidiform mole is a form of gestational trophoblastic disease (GTD) that is characterised by excessive proliferation of the trophoblastic tissue with hydropic swelling of chorionic villi [1]. GTD represents a spectrum of placental trophoblastic disorders, some of which are benign molar pregnancies, and others are malignant, including invasive mole and choriocarcinoma, which necessitates close follow-up using serial serum beta-human chorionic gonadotropin (β-hCG) [2,3].

Hydatidiform moles are classified based on their genetic origin, morphology, and clinical behaviour into complete hydatidiform mole (CHM) and partial hydatidiform mole (PHM) [4,5]. CHMs are associated with a higher risk of persistent trophoblastic disease (approximately 15-20%), whereas PHMs have a much lower risk. In contrast, non-molar hydropic abortions do not have an increased risk of developing trophoblastic neoplasia [2,6]. Therefore, accurate differentiation between CHM, PHM, and non-molar gestations is essential for proper clinical management and follow-up.

Traditionally, the diagnosis is made by histomorphological examination of sections stained with hematoxylin and eosin (H&E). The classical histopathological features of CHM are diffuse villous oedema, cistern formation, circumferential trophoblastic hyperplasia, absence of embryonic tissue, and underdevelopment of villous vasculature, whereas PHM is characterised by the presence of both normal and hydropic villi, focal trophoblastic hyperplasia, and irregular villous contours [6]. However, due to widespread use of ultrasonography and β-hCG testing along with early evacuation of molar pregnancies, these characteristic features may not be fully developed [5,6].

In early gestational specimens, immature chorionic villi may normally appear edematous, making it difficult to distinguish them from molar gestations. Hydropic abortions associated with chromosomal anomalies can resemble molar pregnancies, resulting in marked interobserver variability based solely on histopathologic findings [7]. These diagnostic limitations highlight the need for reliable ancillary techniques to improve diagnostic accuracy.

Immunohistochemistry (IHC) has emerged as a valuable asset in this field. The protein p57KIP2 is encoded by the CDKN1C gene, which plays an important role in the regulation of cell cycle and trophoblastic differentiation. It is a paternally imprinted and maternally expressed protein that serves as a marker of maternal genomic contribution [8]. CHMs are androgenetic with no maternal DNA in the genome and do not express p57 in the villous cytotrophoblasts and stromal cells. In contrast, PHMs and non-molar gestations retain maternal genetic material and demonstrate nuclear positivity [9,10].

Because of this distinctive expression pattern, p57 IHC is a useful diagnostic tool in differentiating CHM from PHM and non-molar gestations, particularly in morphologically ambiguous cases. Incorporation of p57 immunostaining into routine evaluation can improve diagnostic accuracy and guide appropriate clinical management.

Therefore, the present study was undertaken to evaluate the diagnostic utility of p57 IHC in differentiating CHM and PHM from non-molar pregnancies, with emphasis on its role in resolving morphologically ambiguous cases.

## Materials and methods

This hospital-based cross-sectional observational study was conducted in the Department of Pathology at a tertiary referral hospital from March 2024 to October 2025. The study included histopathologically diagnosed cases of CHM, PHM, and non-molar pregnancies received in the Department of Pathology during the study period. A random sampling method was used to enrol cases until the required sample size was attained. Only specimens with adequate tissue for histopathological and immunohistochemical evaluation were included, whereas autolysed specimens were excluded from the study.

All specimens were fixed in 10% buffered formalin and processed using routine histopathological techniques. Paraffin-embedded tissue blocks were prepared, and sections were stained with H&E for histomorphological evaluation. Representative paraffin blocks from each case were selected for p57 IHC. Four-micrometre-thick sections were mounted on charged slides and stained using standard protocols. Antigen retrieval was done using Tris-ethylenediaminetetraacetic acid (Tris-EDTA) buffer, and staining was visualised using a diaminobenzidine (DAB) chromogen system [11,12].

Immunohistochemical evaluation was performed by assessing nuclear expression of p57 in villous cytotrophoblasts and stromal cells. Nuclear staining in these cells was interpreted as positive expression, indicating the presence of maternal genomic material, while absence of staining was considered negative. Syncytiotrophoblasts served as internal negative controls, and maternal decidual stromal cells served as internal positive controls. Based on immunohistochemical findings, cases were classified as p57 positive or p57 negative.

Sample size was calculated using G*Power software (Heinrich-Heine-Universität Düsseldorf, Düsseldorf, Germany). Assuming an anticipated p57 expression of 52% in molar pregnancies and 95% in non-molar pregnancies [13], a minimum sample size of 60 cases (30 per group) was required to achieve a statistical power of 98% at a significance level of 0.05.

Statistical analysis was performed using IBM SPSS Statistics for Windows, Version 29 (Released 2022; IBM Corp., Armonk, New York, United States). Data were expressed as frequencies and percentages. Associations between categorical variables were analysed using the chi-square test or Fisher’s exact test. Sensitivity, specificity, positive predictive value, and negative predictive value were calculated to assess diagnostic performance. A p-value <0.05 was considered statistically significant.

Institutional Ethics Committee (IEC) approval was obtained prior to commencement of the study from BLDE (Deemed to be University), Shri B. M. Patil Medical College, Hospital and Research Centre, Vijayapura, Karnataka, India (approval no.: BLDE(DU)/IEC-SBMPMC/134/2023-24; approval date: 10/02/2024). Written informed consent was obtained from all participants before their inclusion in the study.

## Results

A total of 60 cases of products of conception were included, comprising 30 non-molar pregnancies and 30 molar gestations on initial histopathological evaluation.

Baseline characteristics

The age of patients ranged from 19 to 42 years, with the majority in the 21 to 25 years age group (36.7%, n=22), followed by 26 to 30 years (23.3%, n=14) and 31 to 35 years (20.0%, n=12). The remaining cases were distributed in the age groups 19 to 20 years and 36 to 42 years (10.0% each, n=6).

Histopathological diagnosis

On H&E examination, 30 cases (50.0%) were diagnosed as non-molar pregnancies. Among molar gestations, 14 cases (23.3%) were PHM, 12 cases (20.0%) CHM, and four cases (6.7%) were categorised as hydatidiform mole (unclassified) (Table 1).

**Table 1:** Distribution of study subjects based on histopathological diagnosis (n=60)

Histopathological diagnosis (H&E)	Number of cases	Percentage (%)
Complete hydatidiform mole	12	20.0
Partial hydatidiform mole	14	23.3
Hydatidiform mole (unclassified)	4	6.7
Non-molar pregnancy (products of conception)	30	50.0
Total	60	100.0

Diagnostic reclassification following p57 IHC

Following p57 IHC, the final diagnosis included 30 cases (50.0%) of non-molar pregnancy, 18 cases (30.0%) of CHM, and 12 cases (20.0%) of PHM.

**Figure 1:**
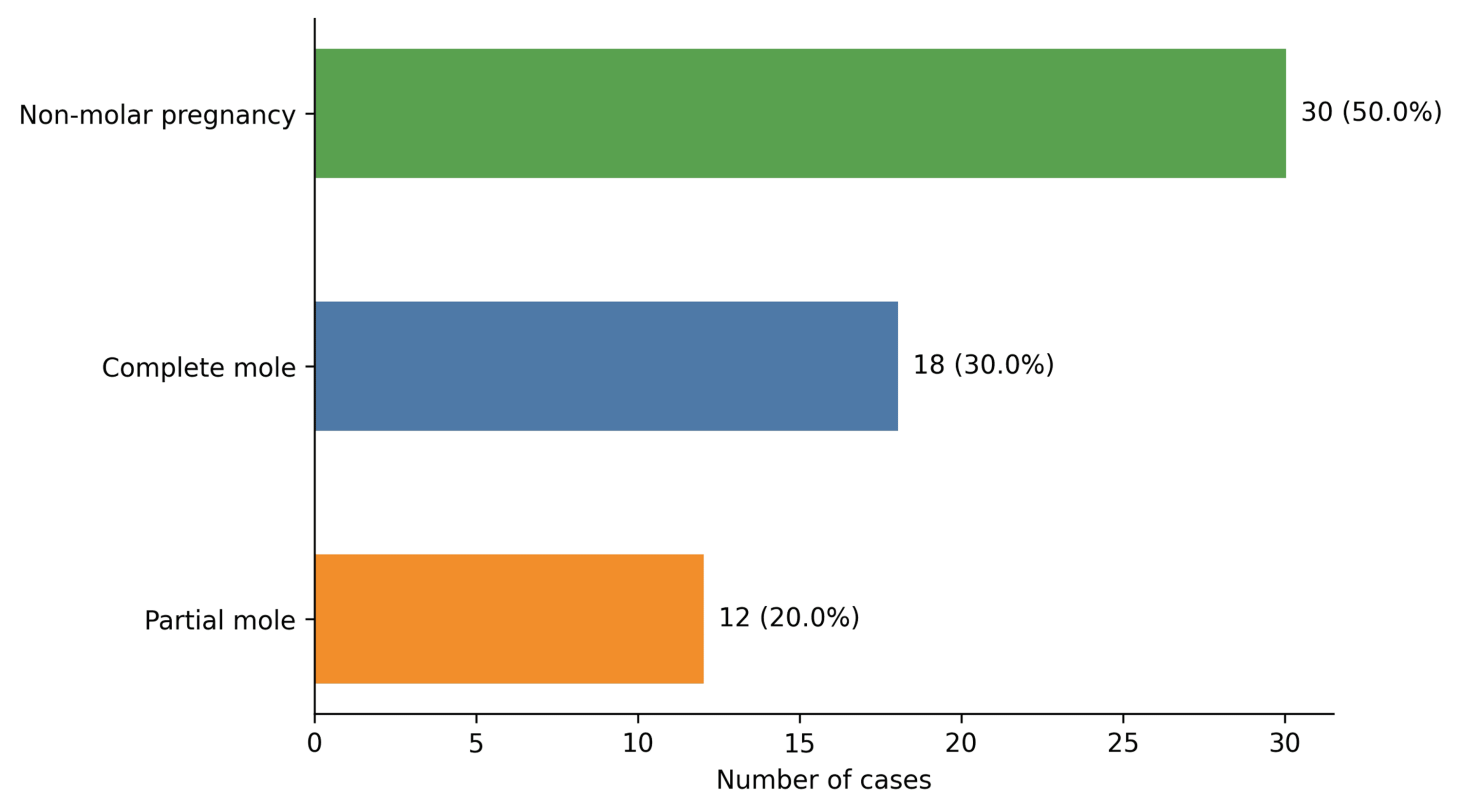
Distribution of cases based on revised diagnosis after p57 immunohistochemistry.

p57 IHC resulted in diagnostic reclassification in 8 of 60 cases (13.3%) (Table 2). All four cases initially categorised as hydatidiform mole (unclassified) were reclassified as CHM. In addition, three cases diagnosed as PHM were reclassified as CHM, while one case initially diagnosed as CHM was reclassified as PHM (Figure 1).

**Table 2:** Revised diagnosis after p57 IHC (n=60) HPR: histopathological report; IHC: immunohistochemistry

Initial HPR diagnosis	Revised diagnosis after IHC	Number of cases
Hydatidiform mole (unclassified)	Complete hydatidiform mole	4
Partial hydatidiform mole	Complete hydatidiform mole	3
Complete hydatidiform mole	Partial hydatidiform mole	1
Total cases with change		8 (13.3%)

Comparison of diagnoses before and after p57 IHC showed a shift in distribution; however, this difference did not reach statistical significance on the Bowker test of symmetry (χ^2^=5.80, df=2, p=0.055) (Table 3).

**Table 3:** Comparison of histopathological diagnosis before and after p57 IHC (Bowker test of symmetry) Bowker test of symmetry: χ^2^=5.80, df=2, p=0.055 IHC: immunohistochemistry; H&E: hematoxylin and eosin

Diagnosis category	Before IHC (H&E)	After IHC
Non-molar pregnancy	30	30
Molar - unclassified	4	0
Complete hydatidiform mole	12	18
Partial hydatidiform mole	14	12

**Figure 2:**
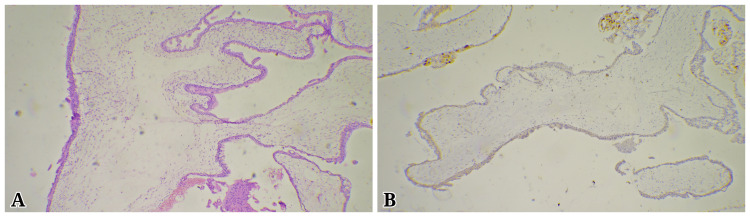
(A) Complete hydatidiform mole showing villous oedema with cistern formation (H&E, ×200); (B) Complete hydatidiform mole showing p57 negativity in cytotrophoblasts and stromal cells of chorionic villi (IHC, ×200) IHC: immunohistochemistry; H&E: hematoxylin and eosin

**Figure 3:**
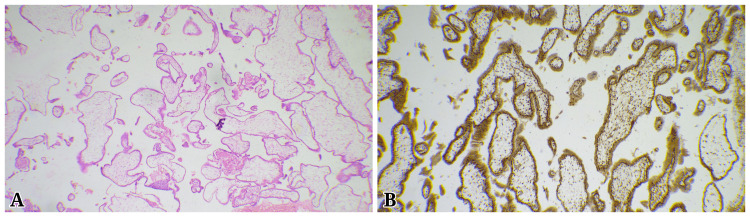
(A) Partial hydatidiform mole showing villous oedema with mild trophoblastic hyperplasia (H&E, ×100); (B) Partial hydatidiform mole showing p57 positivity in cytotrophoblasts and stromal cells of chorionic villi (IHC, ×100) IHC: immunohistochemistry; H&E: hematoxylin and eosin

**Figure 4:**
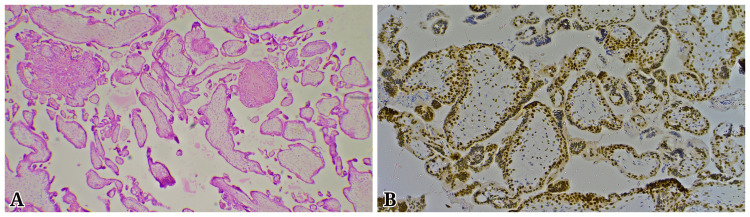
(A) Non-molar pregnancy (products of conception) showing villous oedema involving almost all villi; however, villi are vascularised and do not show trophoblastic hyperplasia (H&E, ×100); (B) Non-molar pregnancy (products of conception) showing p57 positivity in cytotrophoblasts and stromal cells of chorionic villi (IHC, ×200) IHC: immunohistochemistry; H&E: hematoxylin and eosin

Figures 2-4 illustrate the histomorphological and p57 immunohistochemical features of some representative cases. CHM exhibited widespread villous oedema with prominent cistern formation on H&E staining, and a lack of nuclear p57 expression in villous cytotrophoblasts and stromal cells on IHC, as seen in Figure 2. Conversely, PHM exhibited villous oedema along with focal trophoblastic hyperplasia on H&E staining, while maintaining nuclear p57 positivity in cytotrophoblasts and stromal cells on IHC, as seen in Figure 3. Non-molar pregnancies showed villous oedema and had blood vessels, but there was no significant trophoblastic hyperplasia on H&E. Preserved nuclear p57 expression on IHC was seen in Figure 4.

Association between histopathological diagnosis and p57 expression

A statistically significant association was observed between histopathological diagnosis and p57 expression (χ^2^=42.56, df=3, p<0.001).

All non-molar pregnancy cases (30/30) showed positive nuclear p57 expression. All unclassified molar cases (4/4) were p57 negative. Among CHMs, 11 of 12 cases (91.7%) were p57 negative, while one case (8.3%) showed positivity. Among PHMs, 11 of 14 cases (78.6%) were p57 positive, while three cases (21.4%) were negative (Table 4).

**Table 4:** Association between histopathological diagnosis and p57 immunohistochemistry expression Overall association (Pearson chi-square, 4×2): χ^2^=42.56, df=3, p<0.001.

Diagnosis category	p57 positive, n (%)	p57 negative, n (%)	Total	Fisher’s exact p-value
Non-molar pregnancy	30 (100.0)	0 (0.0)	30	<0.001
Molar - unclassified	0 (0.0)	4 (100.0)	4	0.003
Complete hydatidiform mole	1 (8.3)	11 (91.7)	12	<0.001
Partial hydatidiform mole	11 (78.6)	3 (21.4)	14	0.236
Total	41	19	60	-

Diagnostic utility of p57 IHC

All p57-negative cases (n=18) corresponded to CHM (Figure 2), whereas all p57-positive cases (n=42) included PHM (n=12) and non-molar pregnancies (n=30). This demonstrates the utility of p57 IHC in distinguishing CHM from gestations with maternal genomic contribution.

Diagnostic performance of histopathology

The diagnostic performance of histopathological examination was studied using p57 IHC as the reference standard.

In the cases of CHM, histopathology demonstrated a sensitivity of 61.1%, specificity of 91.7%, positive predictive value of 91.7%, and negative predictive value of 61.1%, with an overall diagnostic accuracy of 73.3%. These findings indicate that although histopathology is highly specific for diagnosing CHM, its relatively low sensitivity suggests that some CHMs may be misclassified on morphological assessment alone.

For PHM, histopathology demonstrated a sensitivity of 91.7%, specificity of 83.3%, positive predictive value of 78.6%, and negative predictive value of 93.8%, with an overall diagnostic accuracy of 86.7%. However, the reduced positive predictive value shows that a subset of cases diagnosed as PHM on morphology were subsequently reclassified as CHM following p57 IHC.

Role of p57 in unclassified cases

All four cases which were initially categorised as hydatidiform mole (unclassified) on histomorphological examination were reclassified as CHM after p57 IHC. This finding highlights the limitations of histopathology alone in ambiguous cases and underscores the value of p57 immunostaining in achieving definitive classification of molar pregnancies.

## Discussion

Important findings in this study were the effect of p57 IHC on diagnostic reclassification. In the present study, p57 IHC led to diagnostic revision in eight out of 60 cases (13.3%), including four unclassified hydatidiform moles reclassified as CHM, three PHMs upgraded to CHM, and one case initially diagnosed as CHM reclassified as PHM. This finding demonstrates the proclivity for early CHMs to resemble PHMs morphologically and emphasises the risk of underdiagnosing CHM by relying solely on the histopathology. Such misclassification has important clinical implications, since CHMs have been found to be associated with a much higher risk for persistent GTD and hence require prolonged post-evacuation surveillance [2,3].

Conversely, histopathological diagnosis was highly sensitive for PHM, and the majority of PHMs were therefore correctly identified on routine microscopy. However, specificity and positive predictive value were comparatively lower due to misclassification of some of the CHMs as PHMs. This kind of pattern of diagnostic error has been consistently described in the literature and has been attributed to overlapping villous morphology, focal trophoblastic hyperplasia, and the presence of scalloped villi in early CHMs [9,10]. The high negative predictive value noted in the current study indicates that those cases that were not diagnosed as PHM on histopathology are unlikely to be PHM on final evaluation.

An especially interesting finding from this study was the behaviour of cases which were classified as unclassified hydatidiform mole on first histopathological examination. All such cases were later reclassified as CHM, as performed by IHC for p57, indicating a high predictive association of this category with CHM. Similar findings have been reported in Jacobs et al., in which morphologically ambiguous cases were shown to represent early CHMs with the application of ancillary techniques [14]. This demonstrates the special value of p57 IHC in the resolution of diagnostic ambiguity in this troublesome proportion of cases.

Castrillon et al. were among the first to uncover the usefulness of p57 as a surrogate marker for androgenetic conceptions, reporting a total lack of p57 expression in CHMs and maintenance of p57 expression in PHMs and non-molar gestations [15]. Since then, the reproducibility and diagnostic reliability of p57 IHC have been validated in several studies in different populations, especially in morphologically equivocal cases [10,11,16].

Merchant et al. assessed the use of p57 IHC in a series of morphologically difficult early gestational specimens and described a high concordance between p57 staining and histopathology patterns and final diagnosis, emphasising its usefulness in the resolution of diagnostic dilemmas when histopathology alone was insufficient [10].

Similarly, Popiolek et al. emphasised that p57 IHC is more important as a marker of androgenetic origin, rather than as a marker of molar disease, and the results of p57 should be interpreted along with the histomorphological features [16]. The current study confirms these observations because IHC for p57 was most useful in characterising CHMs and in clarifying some cases of partial or unclassified neoplasms on routine microscopy.

Fukunaga showed a substantial interobserver variability in histopathological diagnosis of early molar pregnancies with frequent disagreements between pathologists even with the application of classical criteria [9]. The relatively low sensitivity of histopathology for CHM seen in the present study is in agreement with these findings and reflects the subtle morphological features of early CHMs, which may lack well-developed cisterns or marked circumferential trophoblastic hyperplasia.

Previous studies have shown that p57 IHC can lead to revision of the initial histopathological diagnosis in a subset of molar pregnancies. Jun et al. reported diagnostic reclassification in 7.1% of cases, where lesions initially interpreted as PHM were later reclassified as CHM after absence of p57 expression [17].

High concordance between histopathological diagnosis and p57 IHC has also been reported. In the present study, concordance was 86.7%, with 13.3% discordance, possibly reflecting the inclusion of diagnostically challenging cases.

Similarly, Mondal et al. demonstrated a significant association between p57 expression and molar classification [12]. In the present study, a highly significant association was also observed (p<0.001), further supporting the utility of p57 IHC in distinguishing CHM from partial mole and non-molar gestations.

Comparison of diagnostic concordance between histopathology and p57 IHC across studies shows high concordance, with discordance rates typically ranging from 6% to 9%. In contrast, the present study demonstrated lower concordance (86.7%) and higher discordance (13.3%), likely due to the inclusion of unclassified cases, where p57 helped resolve diagnostic uncertainty.

Study limitations

This study was conducted at a single tertiary care centre with a relatively small sample size, which may limit the generalisability of the findings. Molecular genotyping, the gold standard for definitive classification of molar pregnancies, could not be performed due to limited resource availability. Therefore, diagnostic categorisation in the present study was based on histomorphological assessment in conjunction with p57 IHC and lacked molecular confirmation. In addition, histopathological evaluation of molar pregnancies may be subject to interobserver variability, particularly in cases with overlapping morphological features. Future multicentric studies involving larger sample sizes and incorporation of molecular genotyping are warranted to further validate these findings and enhance their generalisability.

## Conclusions

To conclude, the present study demonstrates that p57 IHC is a useful adjunct to routine histopathological evaluation in the diagnosis of molar pregnancies. Although histopathology remains the primary diagnostic method, it may have limitations in accurately subtyping molar gestations, particularly in early specimens with overlapping morphological features. The application of p57 immunostaining improves diagnostic accuracy, especially in distinguishing CHM from PHM and non-molar gestations. Reclassification of previously unclassified cases in this study further highlights its diagnostic value. Therefore, the combined use of histopathology and p57 IHC provides a reliable approach for accurate classification and appropriate clinical management of molar pregnancies.
